# Anticoagulation Therapy in Advanced Chronic Kidney Disease: Balancing Bleeding and Thromboembolic Risk

**DOI:** 10.3390/ijms27062577

**Published:** 2026-03-11

**Authors:** Ioana Livia Suliman, Liliana-Ana Tuta, Camelia Pana, Florin Gabriel Panculescu, Andreea Alexandru, Dragos Fasie, Bogdan Cimpineanu, Stere Popescu, Florin-Daniel Enache, Radu Adrian Nitu, Tatiana Chisnoiu, Marius Florentin Popa, Iuliana-Cezara Tudor, Mihaela Lavinia Mihai, Sorin Deacu, Georgeta Camelia Cozaru, Ion Bordeianu

**Affiliations:** 1 Faculty of Medicine, “Ovidius” University of Constanta, 900470 Constanta, Romania; panculescu_i@yahoo.com (I.L.S.); gabriel.panculescu@yahoo.ro (F.G.P.); cimpineanub@yahoo.com (B.C.); stelu_popescu@yahoo.com (S.P.); florin.enache@365.univ-ovidius.ro (F.-D.E.); radu_nitzu@yahoo.com (R.A.N.); tatiana_ceafcu@yahoo.com (T.C.); marius_popa2005@yahoo.com (M.F.P.); sorin.deacu@365.univ-ovidius.ro (S.D.); ion_bordeianu@hotmail.com (I.B.); 2 ”Sfantul Apostol Andrei” Emergency County Clinical Hospital, 900591 Constanta, Romania; dragosfasie@yahoo.com (D.F.); m.mihaelalavinia@yahoo.ro (M.L.M.); 3 Center for Research and Development of the Morphological and Genetic Studies of Malignant Pathology (CEDMOG), “Ovidius” University of Constanta, 900591 Constanta, Romania; drcozaru@yahoo.com; 4 Academy of Romanian Scientist, 3 Ilfov Street, 050044 Bucharest, Romania

**Keywords:** chronic kidney disease, anticoagulation, bleeding, thrombosis, hemodialysis, apixaban

## Abstract

Advanced chronic kidney disease (CKD) presents a complex hemostatic paradox, characterized by a simultaneous increase in bleeding and thromboembolic risks. This study aimed to evaluate and compare the safety and efficacy of apixaban versus acenocoumarol in a real-world cohort of patients across advanced CKD stages (pre-dialysis and hemodialysis). We conducted a retrospective observational study including 84 adults with CKD stages 4 and 5 (40.5% in stage 4; 59.5% in stage 5, of whom 86.0% were on chronic hemodialysis) receiving oral anticoagulation for at least 3 months. Patients were stratified by CKD stage and anticoagulant type (apixaban vs. acenocoumarol). Clinical outcomes included major and clinically relevant bleeding, anticoagulant overdose, and thromboembolic events. Bleeding events were more frequent in CKD stage 5 than in stage 4 (66.0% vs. 44.1%, *p* = 0.06), highlighting a pronounced hemorrhagic burden that increases with disease progression. Apixaban was associated with significantly fewer total bleeding events (44.4% vs. 66.7%, *p* = 0.04) and a lower rate of anticoagulant overdoses (5.6% vs. 18.8%, *p* = 0.04) compared with acenocoumarol. Thromboembolic event rates did not differ significantly between the two anticoagulant groups (13.9% vs. 16.7%, *p* = 0.72). Conventional risk scores (CHA_2_DS_2_-VASc and HAS-BLED) showed limited discriminatory capacity for actual clinical events in this advanced CKD population. In patients with advanced CKD, oral anticoagulation is associated with a high hemorrhagic burden that intensifies as renal function declines. Apixaban demonstrated a more favorable safety profile than acenocoumarol without a loss of thromboembolic protection. These findings suggest that stage-specific biological factors, rather than conventional risk models alone, should guide anticoagulation strategies in this population.

## 1. Introduction

Chronic kidney disease (CKD) is a progressive systemic condition associated with substantial cardiovascular morbidity and mortality, particularly in its advanced stages. Patients with CKD stages 4 and 5 exhibit a markedly increased prevalence of atrial fibrillation and other thromboembolic conditions, frequently necessitating long-term oral anticoagulation therapy. However, the management of anticoagulation in this population represents a major clinical challenge, as progressive renal dysfunction profoundly alters haemostatic balance [1,2].

Advanced CKD is characterized by a complex haemorrhagic–thrombotic paradox, in which bleeding tendency and prothrombotic mechanisms coexist. Uremia-related platelet dysfunction, impaired platelet–endothelial interactions, endothelial injury, chronic inflammation, and anemia contribute to defective primary haemostasis and increased bleeding risk. At the same time, activation of coagulation pathways, endothelial activation, and vascular remodeling sustain a persistent thrombotic potential. This unstable haemostatic equilibrium complicates both risk assessment and therapeutic decision-making [3,4].

Oral anticoagulation further amplifies these challenges. Altered pharmacokinetics and pharmacodynamics in advanced CKD, including reduced renal clearance, changes in protein binding, and interactions with uremic toxins, may lead to unpredictable anticoagulant exposure. Patients are therefore vulnerable to both subtherapeutic anticoagulation effects, with ongoing thromboembolic risk, and excessive anticoagulant effects, with clinically significant bleeding. These difficulties are particularly pronounced in patients with CKD stage 5 undergoing chronic hemodialysis, where additional factors such as vascular access manipulation, systemic anticoagulation during dialysis sessions, and dialysis-related endothelial stress further destabilize haemostasis [5].

Despite the clinical relevance of these issues, evidence guiding anticoagulation strategies in advanced CKD remains limited. Patients with severe kidney impairment and those undergoing dialysis are often excluded from randomized clinical trials of anticoagulant therapy, and existing guidelines rely largely on extrapolated data. Consequently, clinicians must frequently balance competing hemorrhagic and thrombotic risks in the absence of robust, CKD-specific evidence [6,7].

In this context, real-world observational studies are essential to characterize anticoagulation-related outcomes in advanced CKD. Evaluating hemorrhagic and thrombotic complications across CKD stages and anticoagulant regimens may provide valuable insights into the biological and clinical determinants of risk [8].

The present study aimed to investigate the challenges of oral anticoagulation therapy in patients with advanced CKD stages 4 and 5, with a particular focus on the hemorrhagic–thrombotic balance. By analyzing clinical characteristics, laboratory parameters, and anticoagulation-related outcomes in a real-world cohort, this study sought to better define the factors contributing to bleeding and thrombotic events in this biologically complex population.

## 2. Results

### 2.1. Characteristics of the Study Population

A total of 84 patients with advanced CKD receiving oral anticoagulation therapy were included in the analysis. Of these, 34 patients (40.5%) were classified as CKD stage 4, while 50 patients (59.5%) had CKD stage 5. The cohort was further stratified by dialysis status: 43 patients (51.2%) were undergoing chronic hemodialysis (CKD stage 5), whereas 41 patients (48.8%) were not on dialysis.

Baseline demographic characteristics stratified by CKD stage, along with corresponding *p* values, are summarized in Table 1.

### 2.2. Clinical Comorbidities and Cardiovascular Burden

Cardiovascular and systemic comorbidities were highly prevalent in the study population, reflecting the advanced stage of CKD and the substantial baseline cardiovascular risk. Arterial hypertension was the most frequent comorbidity, affecting 95.1% of patients overall, with a similar prevalence in CKD stage 4 and CKD stage 5 patients [9].

Type 2 diabetes mellitus was present in 53.7% of the cohort, without statistically significant differences between CKD stages. Ischemic heart disease and heart failure were also common, affecting 61.0% and 58.5% of patients, respectively, and showed comparable distributions between CKD stage 4 and stage 5 groups [10,11].

A history of cerebrovascular disease, including prior stroke or transient ischemic attack, was documented in 30.5% of patients. Prior myocardial infarction and peripheral arterial disease were observed in 30.5% and 28.0% of the cohort, respectively, with no significant differences between CKD stages. Venous thromboembolic disease was present in 20.7% of patients [12,13].

Chronic liver disease was identified in 13.4% of patients. A history of gastrointestinal bleeding was more frequently reported among CKD stage 5 patients (26.5%) compared with those in CKD stage 4 (18.2%), although this difference did not reach statistical significance. Lifestyle-related factors, including active smoking and alcohol consumption, were similarly distributed between groups [14].

Overall, the burden of cardiovascular and systemic comorbidities was comparable between CKD stage 4 and CKD stage 5 patients, supporting the validity of subsequent analyses evaluating laboratory abnormalities and anticoagulation-related outcomes. Clinical comorbidities stratified by CKD stage are summarized in Table 2 [15,16,17].

### 2.3. Baseline Laboratory Parameters

Baseline laboratory evaluation revealed significant differences between CKD stage 4 and CKD stage 5 patients, reflecting the progressive biological impact of advanced renal dysfunction. Patients with CKD stage 5 exhibited more severe anemia compared with those in CKD stage 4, with significantly lower hemoglobin levels (9.67 ± 1.53 vs. 10.50 ± 1.29 g/dL, *p* = 0.01). Lymphocyte counts were also significantly reduced in CKD stage 5 patients (1.00 ± 0.48 vs. 1.42 ± 0.51 × 10^3^/µL, *p* < 0.001), suggesting increased immune dysregulation [18,19].

Markers of renal function demonstrated the expected stage-dependent deterioration. Serum urea and creatinine levels were significantly higher in CKD stage 5, while estimated glomerular filtration rate was markedly lower compared with CKD stage 4 patients (all *p* < 0.001). Nutritional status was adversely affected in advanced disease, as reflected by significantly lower serum albumin and total protein levels in CKD stage 5 patients [20].

Electrolyte disturbances were more pronounced in CKD stage 5, particularly hyperkalemia and hypocalcemia, both of which reached statistical significance. In contrast, serum sodium levels did not differ significantly between groups. Conventional coagulation parameters, including INR and aPTT, were comparable between CKD stages [20,21].

Cardiovascular and coagulation-related biomarkers showed significant stage-dependent increases. D-dimer and B-type natriuretic peptide (BNP) levels were significantly higher in CKD stage 5 patients, indicating enhanced thrombo-inflammatory activity and increased cardiovascular stress [22,23].

Baseline laboratory parameters stratified by CKD stage are summarized in Table 3.

Following the assessment of baseline characteristics and laboratory parameters, the clinical efficacy and safety outcomes were evaluated. Hemorrhagic complications were more frequent than thrombotic events in the overall study population, highlighting the increased bleeding vulnerability associated with advanced kidney disease under oral anticoagulation therapy.

The cumulative incidence of any bleeding event was higher in CKD stage 5 compared to CKD stage 4 (66.0% vs. 44.1%, *p* = 0.06). When stratified by anticoagulant type, patients treated with apixaban exhibited a significantly lower rate of total bleeding events (44.4% vs. 66.7%, *p* = 0.04) and a reduced incidence of anticoagulant overdose (5.6% vs. 18.8%, *p* = 0.04) compared to those receiving acenocoumarol. Major bleeding and upper gastrointestinal bleeding remained uncommon across all groups.

Thromboembolic protection was comparable between the two treatment strategies, with any thromboembolic event recorded in 15.5% of the total cohort. Vascular access thrombosis occurred predominantly in CKD stage 5 patients, while ischemic stroke and venous thromboembolic events were observed at low and comparable rates regardless of the anticoagulant type or CKD stage.

The comprehensive comparison of clinical outcomes, stratified by both CKD stage and anticoagulant type, is summarized in Table 2.

## 3. Discussion

This study provides a biologically grounded perspective on oral anticoagulation in advanced kidney disease, highlighting the marked predominance of hemorrhagic over thrombotic complications in patients with CKD stages 4 and 5. Our findings underscore a critical paradox: as kidney function declines, the risk of bleeding increases disproportionately compared to the risk of thromboembolism, creating a narrow and precarious therapeutic window. In our cohort, gingival bleeding emerged as the most frequent hemorrhagic manifestation, affecting 50% of the total population. This highlights the vulnerability of primary haemostasis, where uremic toxins interfere with von Willebrand factor-mediated platelet adhesion and subsequent aggregation.

The observed increase in bleeding complications with CKD progression parallels the worsening of anemia and hypoalbuminemia. From a mechanistic standpoint, these alterations reflect a convergence of uremia-related platelet dysfunction and chronic inflammatory stress. Anemia contributes to this risk by reducing the rheological displacement of platelets toward the vessel wall, further impairing primary haemostatic plug formation. Moreover, hypoalbuminemia in advanced CKD is not merely a nutritional marker but a surrogate for chronic inflammation and endothelial activation. Reduced albumin levels may alter the free fraction of highly protein-bound drugs, such as vitamin K antagonists, potentially explaining the higher rates of over-anticoagulation observed in our CKD stage 5 subgroup.

The comparison between anticoagulant strategies (Table 4) revealed a significantly more favorable safety profile for apixaban compared with acenocoumarol. The lower incidence of total bleeding (44.4% vs. 66.7%) and anticoagulant overdose (5.6% vs. 18.8%) in the apixaban group is clinically significant. This superiority can be attributed to the pharmacological stability of Factor Xa inhibitors. VKAs like acenocoumarol are notoriously difficult to manage in uremic environments due to dietary fluctuations, altered cytochrome P450 activity, and the frequent need for antibiotic co-administration in dialysis patients, all of which destabilize the International Normalized Ratio (INR). In contrast, apixaban’s minimal renal clearance (approximately 27%) and lack of significant interference with uremic metabolites provide a more “predictable” anticoagulant effect, even in the presence of severely reduced eGFR.

Notably, thrombotic events, including vascular access thrombosis and ischemic stroke, remained relatively infrequent and did not demonstrate consistent stage-dependent differences. This supports the concept that advanced CKD represents a state of “unstable haemostatic equilibrium” rather than simple hypocoagulability. The presence of elevated D-dimer and BNP levels, especially in stage 5 patients, confirms that prothrombotic pathways remain active despite the clinical predominance of bleeding. This “uremic prothrombotic milieu” is driven by increased tissue factor expression and impaired endogenous fibrinolysis, which may explain why thrombotic protection must be maintained even when the bleeding risk is high.

Several limitations should be acknowledged. The retrospective, single-center design limits causal inference and the sample size restricts the detection of differences in less frequent thrombotic events. Furthermore, the laboratory profiling followed institutional protocols. Nevertheless, the strengths of this study include the inclusion of a well-characterized high-risk population and the use of real-world data, which are essential in a field where randomized controlled trials often exclude patients with eGFR < 15 mL/min/1.73 m^2^. Our results provide a necessary bridge between theoretical pharmacological benefits and clinical reality in one of the most challenging patient populations in nephro-cardiology.

## 4. Materials and Methods

### 4.1. Study Design and Population

This retrospective, observational, single-center study included adult patients with advanced CKD who received oral anticoagulation therapy and were followed in the Department of Nephrology, “Sf. Apostol Andrei” Constanța County Emergency Hospital, Romania. he study evaluated adult patients with advanced CKD receiving oral anticoagulation therapy over a 4-year period between 1 January 2021 and 31 December 2024.

Eligible patients were aged ≥18 years and had documented CKD stage 4 or stage 5, including both pre-dialysis patients and those undergoing chronic hemodialysis. All patients had received continuous oral anticoagulation therapy for at least three months prior to inclusion for standard clinical indications, including atrial fibrillation or previous thromboembolic events.

Patients were stratified according to CKD stage at baseline into:

- CKD stage 4 (pre-dialysis);
- CKD stage 5 pre-dialysis;
- CKD stage 5D (End Stage Renal Disease on chronic hemodialysis).

Due to the retrospective design and the use of existing electronic medical records, blinding was not applicable. Data extraction and verification were performed by investigators not involved in direct patient care to minimize selection and information bias.

The primary indication for long-term oral anticoagulation in our study population was non-valvular atrial fibrillation (NVAF), which was present in all 84 patients (100%). Additionally, a significant proportion of the cohort had secondary indications, including deep vein thrombosis (*n* = 17, 20.7%) and pulmonary embolism (*n* = 14, 17.1%).

### 4.2. Inclusion and Exclusion Criteria

**Inclusion criteria:**

- Age ≥ 18 years
- Advanced CKD (stage 4 or 5), defined as estimated glomerular filtration rate (eGFR) < 30 mL/min/1.73 m^2^, calculated using the CKD-EPI 2021 equation [24]
- Treatment with oral anticoagulation therapy for a minimum of three months
- Documented indication for anticoagulation (atrial fibrillation, venous thromboembolism, or previous thromboembolic event)

**Exclusion criteria:**

- Age > 80 years at the time of inclusion;
- History of kidney transplantation;
- Treatment with peritoneal dialysis;
- Severe anemia, defined as hemoglobin level < 7 g/dL;
- Presence of active bleeding, documented clinically or by imaging methods, at the time of hospitalization or cohort inclusion;
- Hospitalization within the previous 30 days for severe infections;
- Active malignancy or ongoing systemic immunomodulatory therapy;
- Incomplete clinical or laboratory data preventing adequate analysis.

### 4.3. Data Collection and Clinical Parameters

- Demographic and clinical data, including age, sex, comorbidities (hypertension, diabetes mellitus, ischemic heart disease, heart failure, prior stroke or transient ischemic attack), dialysis status, and duration of kidney disease, were extracted from electronic medical records.
- Laboratory parameters collected at baseline included hemoglobin, platelet count, serum creatinine, eGFR, serum urea, albumin, electrolytes, and coagulation parameters, including international normalized ratio (INR) where applicable. Inflammatory and biochemical markers reflecting nutritional and inflammatory status were also recorded.
- Stroke and bleeding risk were assessed using the CHA_2_DS_2_-VASc and HAS-BLED scores [25,26].
- Bleeding events were classified according to the International Society on Thrombosis and Haemostasis (ISTH) criteria as major bleeding, clinically relevant non-major bleeding, or overdose-related bleeding [27].

### 4.4. Outcomes

**Primary Outcomes**

- Major bleeding events;
- Thromboembolic events, including ischemic stroke, systemic embolism, deep vein thrombosis, and pulmonary embolism.

Major bleeding was defined according to the International Society on Thrombosis and Haemostasis (ISTH) criteria.

**Secondary Outcomes**

- Clinically relevant non-major bleeding;
- Bleeding-related hospitalization;
- Need for blood transfusion;
- Discontinuation of anticoagulation therapy;
- All-cause mortality.

All outcomes were identified through review of hospital records, discharge summaries, laboratory results, and imaging reports. Events were validated by two investigators independently to minimize information bias.

**Anticoagulant overdose was defined as follows:**

- **For acenocoumarol:** An International Normalized Ratio (INR) value > 3.0.
- **For apixaban:** The administration of a 5 mg twice-daily dose in patients who met the standard criteria for dose reduction to 2.5 mg twice daily (based on age, weight, and serum creatinine levels) or in those undergoing chronic hemodialysis [28,29].

### 4.5. Statistical Analysis

Statistical analysis was performed using SPSS Statistics (Version 26.0). The normality of data distribution was assessed using the Shapiro–Wilk test. Continuous variables are expressed as mean ± standard deviation (SD) for normally distributed data, or median and interquartile range (IQR) for non-normally distributed variables. Categorical variables are presented as frequencies and percentages.

Comparisons between groups were performed using the *t*-test or Mann–Whitney U test for continuous variables, and the Chi-square or Fisher’s exact test for categorical variables. Event rates were calculated per 100 patient-years. A *p*-value < 0.05 was considered statistically significant.

### 4.6. Ethical Considerations

The study protocol was reviewed and approved by the **Ethics Committee of the Clinical Emergency Hospital of Constanța County (Decision No. 55437/25.08.2025)** and conducted in accordance with the principles of the Declaration of Helsinki. Written informed consent was obtained from all participants prior to inclusion [30].

## 5. Conclusions

Anticoagulation therapy in advanced stages of kidney disease represents a major clinical challenge, arising from a profoundly unstable hemorrhagic–thrombotic balance. Our findings indicate that CKD stage itself is a central determinant of safety, independent of conventional risk profiles. As kidney function deteriorates, the equilibrium between therapeutic efficacy and adverse outcomes becomes increasingly fragile, exposing patients to heightened bleeding vulnerability while thromboembolic risk remains persistent.

The results emphasize the necessity of a stage-adapted, individualized anticoagulation strategy. In this context, the superior safety profile of apixaban over acenocoumarol in our cohort suggests that direct oral anticoagulants may mitigate some of the inherent risks associated with advanced uremia. In the absence of robust randomized evidence for this specific population, real-world data are essential to refine clinical judgment and optimize the management of patients with advanced CKD, in whom maintaining the hemorrhagic–thrombotic balance remains a priority.

## Figures and Tables

**Table 1:** Baseline Demographic Characteristics According to CKD Stage.

Characteristic	CKD Stage 4 (*n* = 34)	CKD Stage 5 (*n* = 50)	Total (*n* = 84)	*p*-Value
Female sex, *n* (%)	15 (44.1%)	23 (46.0%)	38 (45.23%)	0.032
Male sex, *n* (%)	19 (55.8%)	27 (54.0%)	46 (54.76%)	0.043
Urban residence, *n* (%)	23 (67.64%)	31 (62.0%)	54 (64.28%)	0.6
Rural residence, *n* (%)	11 (32.3%)	19 (38.0%)	30 (35.71%)	0.4
Dialysis (G5), *n* (%)	—	43 (86.0%)	43 (51.2%)	—
Pre-dialysis (G5), *n* (%)	—	7 (14.0%)	7 (8.3%)	—

**Table 2:** Clinical Comorbidities According to CKD Stage.

Comorbidity	CKD Stage 4 (*n* = 34)	CKD Stage 5 (*n* = 50)	Total (*n* = 84)	*p*-Value
Arterial hypertension	31 (93.9%)	47 (95.9%)	78 (95.1%)	1.00
Dyslipidemia	19 (57.6%)	26 (53.0%)	45 (54.9%)	0.72
Atrial fibrillation	33 (100%)	49 (100%)	82 (100%)	—
Ischemic heart disease	20 (60.6%)	30 (61.2%)	50 (61.0%)	1.00
Heart failure	18 (54.5%)	30 (61.2%)	48 (58.5%)	0.71
Prior ischemic stroke	11 (33.3%)	14 (28.6%)	25 (30.5%)	0.83
Prior myocardial infarction	9 (27.3%)	16 (32.6%)	25 (30.5%)	0.78
Peripheral arterial disease	8 (24.2%)	15 (30.6%)	23 (28.0%)	0.70
Deep vein thrombosis	6 (18.2%)	11 (22.4%)	17 (20.7%)	0.85
Pulmonary embolism	5 (15.2%)	9 (18.4%)	14 (17.1%)	0.94
Chronic liver disease	4 (12.1%)	7 (14.3%)	11 (13.4%)	1.00
History of bleeding	6 (18.2%)	8 (16.3%)	14 (17.1%)	0.84
Active smoking	12 (36.4%)	17 (34.7%)	29 (35.4%)	1.00
Alcohol consumption	8 (24.2%)	11 (22.4%)	19 (23.2%)	1.00

**Table 3:** Baseline Laboratory Parameters According to CKD Stage.

Parameter	CKD Stage 4 (*n* = 34)	CKD Stage 5 (*n* = 50)	Total (*n* = 84)	*p*-Value
Hemoglobin (g/dL)	10.50 ± 1.29	9.67 ± 1.53	10.00 ± 1.48	0.01
Mean corpuscular volume (fL)	91.67 ± 4.04	92.56 ± 7.23	92.20 ± 6.10	0.54
Leukocytes (×10^3^/µL)	6.21 ± 1.84	6.48 ± 2.11	6.37 ± 2.00	0.58
Lymphocytes (×10^3^/µL)	1.42 ± 0.51	1.00 ± 0.48	1.17 ± 0.52	<0.001
Neutrophils (×10^3^/µL)	4.32 ± 1.37	5.50 ± 4.95	5.02 ± 3.89	0.18
Platelets (×10^3^/µL)	212 ± 64	198 ± 71	204 ± 68	0.36
INR	2.41 ± 0.89	2.68 ± 1.14	2.57 ± 1.05	0.27
aPTT (seconds)	34.2 ± 6.3	36.7 ± 7.9	35.7 ± 7.4	0.14
Urea (mg/dL)	142.6 ± 35.8	196.3 ± 48.7	174.7 ± 49.3	<0.001
Serum creatinine (mg/dL)	3.1 ± 0.7	6.4 ± 1.9	5.1 ± 2.1	<0.001
eGFR (mL/min/1.73 m^2^)	20.78 ± 4.69	9.31 ± 2.97	13.90 ± 6.48	<0.001
Serum albumin (g/dL)	3.60 ± 0.40	3.20 ± 0.50	3.36 ± 0.49	<0.001
Total proteins (g/dL)	6.42 ± 0.61	5.98 ± 0.72	6.15 ± 0.71	0.01
Sodium (mmol/L)	137.8 ± 3.9	136.1 ± 4.6	136.8 ± 4.4	0.09
Potassium (mmol/L)	4.8 ± 0.6	5.3 ± 0.7	5.1 ± 0.7	<0.001
Calcium (mg/dL)	8.6 ± 0.7	8.1 ± 0.8	8.3 ± 0.8	0.01
D-dimers (ng/mL)	842 ± 311	1124 ± 486	1011 ± 459	0.01
BNP (pg/mL)	486 ± 215	912 ± 438	743 ± 432	<0.001

**Table 4:** Clinical Outcomes and Complications Stratified by CKD Stage and Anticoagulant Type.

Outcome	CKD Stage 4 (*n* = 34)		CKD Stage 5 (*n* = 50)		Total (*n* = 84)	*p*-Value
	Apixaban	Acenocumarol	Apixaban	Acenocumarol		
Any bleeding event, *n* (%)	6 (17.6%)	9 (26.5%)	10 (20.0%)	23 (46.0%)	48 (57.1%)	0.04
- Gingival bleeding	5 (14.7%)	9 (26.5%)	9 (18.0%)	19 (38.0%)	42 (50.0%)	0.048
- Major bleeding (ISTH)	0 (0%)	1 (2.9%)	1 (2.0%)	2 (4.0%)	4 (4.8%)	0.64
- Upper GI bleeding	0 (0%)	1 (2.9%)	0 (0%)	2 (4.0%)	3 (3.6%)	0.09
Anticoagulant overdose	1 (2.9%)	2 (5.9%)	1 (2.0%)	7 (14.0%)	11 (13.1%)	0.04
Any thromboembolic event	2 (5.9%)	2 (5.9%)	3 (6.0%)	6 (12.0%)	13 (15.5%)	0.72
- Vascular access thrombosis	1 (2.9%)	0 (0%)	1 (2.0%)	5 (10.0%)	7 (8.3%)	0.48
- Ischemic stroke	1 (2.9%)	1 (2.9%)	1 (2.0%)	2 (4.0%)	5 (6.0%)	1.00
- Venous thromboembolism	0 (0%)	2 (5.9%)	1 (2.0%)	2 (4.0%)	5 (6.0%)	1.00

## Data Availability

The data presented in this study are available upon request from the corresponding author.
